# Identification of Distributed Denial of Services Anomalies by Using Combination of Entropy and Sequential Probabilities Ratio Test Methods

**DOI:** 10.3390/s21196453

**Published:** 2021-09-27

**Authors:** Basheer Husham Ali, Nasri Sulaiman, Syed Abdul Rahman Al-Haddad, Rodziah Atan, Siti Lailatul Mohd Hassan, Mokhalad Alghrairi

**Affiliations:** 1 Department of Electrical and Electronic Engineering, Faculty of Engineering, Universiti Putra Malaysia, Serdang 43400, Malaysia or basheer.husham@aliraqi.edu.iq (B.H.A.); mokhalad.khalel@alkadhum-col.edu.iq (M.A.); 2 Department of Computer Engineering, Al-Iraqia University, Baghdad 10054, Iraq; 3 Department of Computer and Communication Systems Engineering, Faculty of Engineering, Universiti Putra Malaysia, Serdang 43400, Malaysia; sar@upm.edu.my; 4 Department of Software Engineering and Information Systems, Faculty of Computer Science and Information Technology, Universiti Putra Malaysia, Serdang 43400, Malaysia; rodziah@upm.edu.my; 5 Faculty of Electrical Engineering, Universiti Teknologi MARA, Shah Alam 40450, Malaysia; sitilailatul@uitm.edu.my; 6 Department of Computer Techniques Engineering, Imam Al kadhum College (IKC), Baghdad 10087, Iraq

**Keywords:** distributed denial of services attack, entropy, sequential probability ratio test, confusion matrix

## Abstract

One of the most dangerous kinds of attacks affecting computers is a distributed denial of services (DDoS) attack. The main goal of this attack is to bring the targeted machine down and make their services unavailable to legal users. This can be accomplished mainly by directing many machines to send a very large number of packets toward the specified machine to consume its resources and stop it from working. We implemented a method using Java based on entropy and sequential probabilities ratio test (ESPRT) methods to identify malicious flows and their switch interfaces that aid them in passing through. Entropy (E) is the first technique, and the sequential probabilities ratio test (SPRT) is the second technique. The entropy method alone compares its results with a certain threshold in order to make a decision. The accuracy and F-scores for entropy results thus changed when the threshold values changed. Using both entropy and SPRT removed the uncertainty associated with the entropy threshold. The false positive rate was also reduced when combining both techniques. Entropy-based detection methods divide incoming traffic into groups of traffic that have the same size. The size of these groups is determined by a parameter called window size. The Defense Advanced Research Projects Agency (DARPA) 1998, DARPA2000, and Canadian Institute for Cybersecurity (CIC-DDoS2019) databases were used to evaluate the implementation of this method. The metric of a confusion matrix was used to compare the ESPRT results with the results of other methods. The accuracy and f-scores for the DARPA 1998 dataset were 0.995 and 0.997, respectively, for the ESPRT method when the window size was set at 50 and 75 packets. The detection rate of ESPRT for the same dataset was 0.995 when the window size was set to 10 packets. The average accuracy for the DARPA 2000 dataset for ESPRT was 0.905, and the detection rate was 0.929. Finally, ESPRT was scalable to a multiple domain topology application.

## 1. Introduction

With the rapid development of technology, new devices are being connected through the internet each day. Many companies, organizations, universities, hospitals, banks, government units, and other associations have become dependent on computer technology to serve their needs through the internet. However, attackers exploit vulnerabilities that are available in devices, networking connections, or applications to carry out their malicious activities. Cyber-attacks are very dangerous when they are appropriately applied. These kinds of activities can be used to destroy, steal, or breach sensitive information belonging to public or private institutions. These activities can also be used to gain unauthorized access and important data. Many kinds of malicious activities can be performed in computer networks. One of these is a distributed denial of services (DDoS) attack [1].

**Figure 1:**
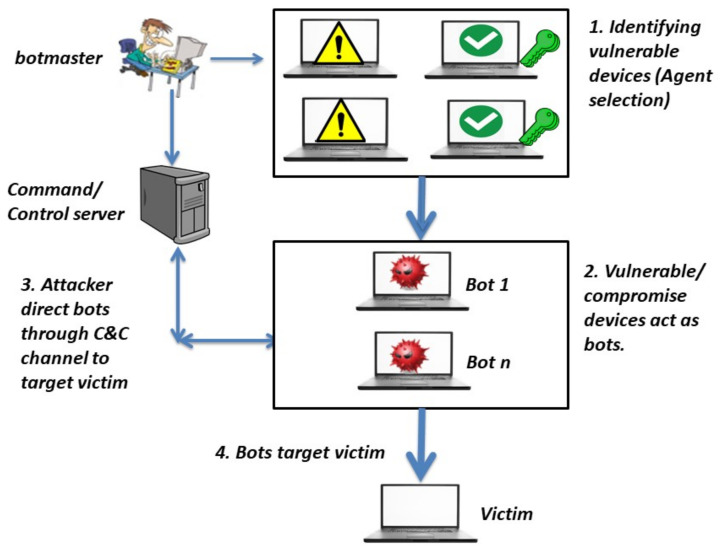
Preparing a botnet for a DDoS attack.

The detection of DDoS attacks is very challenging [2]. DDoS attacks are kind of cyber-attack that target a specific machine or server and lead them to stop providing their services to the devices that are connected to this machine. Attackers in DDoS attacks can form a botnet [1,3]. A botnet is a very large number of malicious devices that are called bots. All of these devices are controlled by a main attacker called a botnet master. A botnet master is responsible for choosing and detecting these compromised devices. Attackers carry out four steps in order to form a botnet. These steps involve identifying vulnerable devices, compromising agents to act as bots, using a C&C channel between the attacker and the bots, and targeting victim using the bots, as shown in Figure 1.

Attackers identify all the expected vulnerable machines in the network and induce or direct them to forward attacked packets or flows toward a specific machine or server. These vulnerabilities can be discovered by means of tools or techniques such as a worm, backdoor, or Trojan horse. They can be identified by sending an email containing a malicious code, such as a virus [4]. This leads to the infection of the machines in the network to create what is called zombies or agents. In turn, zombies can find other vulnerable devices in the network to expand the number of attack teams. The main attacker or bot master can communicate and manage these zombies by using protocols. They obey the attacker’s orders through the command and control (C&C) server [5].

Furthermore, all of these zombies send malicious packets toward the server with the incitement of attacker, whereas the real attacker uses a spoofed IP in order to hide his identity and slow down his discovery. The attacker with its agents sends a very large number of low-rate packets or flows toward the targeted victim. This leads to the server being overloaded with useless packets and prevents legitimate users from getting services [6]. It is similar to unexpected cars or trucks crossing over a highway, which prevents normal cars from passing over the road and causing a traffic jam.

Moreover, a DDoS attacks lead to increase in the processing rate on the targeted side. One way to do this is by exhausting the targeted machine resources, such as CPU, bandwidth, address bus, data bus, control bus, RAM, socket, or hard drive bandwidth. The attackers who targeted the SCO Group website attempted to exhaust the connection of legal users by consuming connection network resources, bandwidth, or switching process capacity [7].

In addition, amplification or reflection is another form of DDoS attack. The attacker sends requested traffics to its zombies, then zombies convert or amplify the received traffic. The converted traffic is larger in size than the original traffic, and they then send this to the victims. Smurf, NTP, DNS amplification, and Fraggle attacks are all examples of these kinds of methods [6].

Moreover, E-business or other large companies are the biggest losers from these kinds of attacks because they lose large amounts of money if users cannot reach their services [8]. For example, Yahoo faced its first DDoS attacks in February 2000. These attacks brought Yahoo’s services down for almost two hours, which led to a decrease in its revenue [7]. Attackers targeted the SCO Group website in 2004 using DDoS attacks, leading it to stop its services. These attacks happened because the system was vulnerable to the Mydoom virus. This virus contained a program that allowed a very large number of machines to target the SCO main computer at the same time. This virus was also retargeted at bank units and public news websites in the United Stated of America and South Korea in 2009. DDoS attacks also launched popular financial websites such as post-finance, master card, PayPal, and Visa card websites. In 2012, popular bank websites in the USA, such as PNC bank, Bank of America, Capital One bank, Fifth Third bank, and Citigroup bank were affected by DDoS attacks. Famous electronic shopping companies have also been exposed to DDoS attacks, such as Amazon, e-bay, and Buy.com [7].

According to Akamai, gaming industries have struggled with DDoS attacks. Akamai noted almost three thousand unique DDoS attacks in the gaming industry from July 2019 till June 2020, making it the largest target for DDoS attacks among other industries. This situation was worsened during COVID-19 lockdowns [9].

The contribution of the paper can be summarized in three points. First, we implement a combination of entropy and SPRT (ESPRT) methods developed to detect DDoS flows and determine infected switch interfaces using Java. Second, we evaluate the detection model and compare it with other detection models using confusion matrix metrics and several datasets, specifically, the DARPA 1998, DARPA 1999, DARPA 2000, and CIC-DDoS2019 datasets. Thirdly, we tested the scalability of the ESPRT and compared it with other detection models. The rest of the paper is organized as follows: related works are discussed in the second section, methodology in the third section, results in the fourth section, and conclusions in the last section.

## 2. Related Works

There are many kinds of techniques that have been adopted to detect a DDoS attack in its early stage. These methods can be categorized into two main groups—application- and network-based detection. Application-based detection is based on monitoring and controlling DDoS attacks in the user application layer, whereas network-based detection is based on monitoring attacks by using network protocols in different layers [10]. Network-based detection can be classified into two methods: the signature-based method and the anomaly-based method. The signature-based method depends on previously identifying known attack patterns and comparing new samples with saved patterns to discover a match or known attacks. This method needs to be updated by adding new patterns of attacks, and it is not able to detect new patterns that are not available in this method [6].

Anomaly-based detection assumes that there is a known model for the benign behavior for the system and any deviation from that behavior is considered malicious. It explains the benign behavior for certain information and performs a deducible test to detect unknown patterns or anomalies [11]. Anomaly-based detection involves offline and online detection methods. Offline detection methods identify attacks after they have occurred, whereas online detection methods identify attacks at the beginning of the attack’s occurrence. Anomaly-based detection can be categorized into many types, such as statistical approaches, data mining, machine learning, deep learning, or combining more than one technique [4,6].

### 2.1. Statistical-Based Detection Techniques

Statistical approaches are a type of mathematical model. They search to find common connections between one or more non-random and/or random variables. The connections of variables are used to predict the results. Statistical approaches to the detection of anomalies provide a deeper analysis per packet in computer networks. They enable the observation and analysis of information per specific time in a very fast time [6]. A number of studies have used these techniques to detect DDoS attacks. In this subsection, related works that have used entropy-based detection and the combination of entropy and other methods are explained and described in this subsection. The entropy approach is a common way to generate features that can be used to classify network flows. These features can be extracted based on calculating entropy for packet fields such as source IP, destination IP, source port, destination port, protocol name, etc. Recently, it has been widely used in the detection of DDoS attacks. Entropy calculation is used to discover randomness in network traffic. If entropy values are high, packets are random and, vice versa, when entropy values are low, packets are not random. The randomness of packets is an indicator of benign behavior, as stated in [12]. The entropy-based detection approach is better than other methods in identifying DDoS attacks for many reasons. It involves simple calculations. It also provides a high detection rate, a low fall-out rate, and high accuracy [13].

Abigail et al. proposed a method to extract many features from packets and to calculate entropy for these features in order to increase accuracy and recall [13]. Ma and Chen presented a method to detect anomalies using entropy chaos analysis and the Lyapunov exponent [14]. This method has two steps. First, they monitor the source and destination IP for incoming packets and calculate the Tsallis entropy of these two fields. In general, the entropy-based method relies on the calculated values of the traffic fields and ignores relationships between each field. This is why the authors proposed a second step, which uses the Lyapunov exponent. The Lyapunov exponent is used to determine the rate of distribution of the two trajectories. Finally, the authors validated their method using the MIT dataset; they found that their model had a TPR of 98.56 and an FPR of 0.42 FPR [14].

Hoque et al. proposed a method based on statistics called the Feature Feature Score (FFS) method. This method has two benefits. The first is its ability to identify attacks and benign traffic. The second is its ability to distinguish between normal and low-rate traffic. This method has three steps. First, features from normal traffic, such as packet rates, the entropy of the source IP, and the diversity of the source IP, are extracted and stored to form a normal profile. Second, the same calculations from the first step are carried out for new incoming packets. Third, the method is used to find similarities and dissimilarities between the normal profile generated in step one and the profile generated in the second step, using a deviation vector and the mean of the extracted features. If the value of the third step is larger than a specific threshold, then the observed traffic is normal; otherwise, there is an attack. Normal and low rates can be distinguished by using a standard deviation vector that defines a variation in the extracted features for a specific sample. The normal samples have a predicted number of traffics, whereas attack samples have an unpredicted number of samples [15]. Linhai and Xiao in [16] used both entropy and SPRT to detect DDoS attacks in software-defined network (SDN) environments. They tested the model using data that were generated using the Scapy tool. They found that their model needs further adjustment in order to prevent or eliminate false positive rates.

### 2.2. Data Mining-Based Detection Techniques

Data mining is a process of reproducing new and useful patterns from a very large amount of data. It can be performed through human intrusion using algorithms and programming tools. It helps to make unknown collected data more usable and clearer. Data mining techniques can be used also to detect DDoS anomalies. For example, Bista and Chitrakar suggested a new approach to identify DDoS attacks based on data mining techniques. They used a clustering method called a heuristics clustering algorithm that is unsupervised learning and followed by a classification method, the naive Bayes method. They used naive Bayes because some malicious traffic contains large numbers of benign packets that cannot be identified using only the heuristics clustering algorithm. Thus, the authors used both methods to handle this issue. They also used multiple datasets in order to validate their proposed method, such as DARPA 2000 and the CAIDA UCSD DDoS Attack 2007 dataset. They used some confusion matrix metrics in order to evaluate their system as well, such as accuracy, the false positive rate, and the true positive rate. Finally, they claimed that their system has good accuracy and a low false positive rate [17].

### 2.3. Machine Learning-Based Detection Techniques

Machine learning involves the use of algorithms and techniques to train machines with historical datasets in order to perform a prediction analysis or classification on a new dataset. Machine learning techniques can be used in the detection of DDoS attacks. For example, Polat et al. proposed a method of detecting DDoS attacks in software-defined networks (SDNs). They extracted important features from SDNs for datasets with and without DDoS attacks and stored results in a new dataset. Then, they tested the new dataset (with features) and the old dataset (without features) on multiple different methods of machine learning to discover their detection ability. They used naive Bayes (NB) and K-nearest neighbors (KNN) classification models, and support vector machine (SVM) and artificial neural network (ANN) methods. Based on their experiment results, they found that the KNN classifier had the highest detection results, at approximately 98.3% [18]. Joao et al. in [19] proposed a novel method to identify DDoS anomalies based on two phases. The first phase is filtering out the mean values of popular features from the data using higher order singular value decomposition (HOSVD). The output of the first step was used as an input for machine learning techniques in order to make a decision about attack availability. They used the CICDDoS2019 and CICIDS2017 Datasets in their evaluation, and found that the accuracy for their proposed detection method was almost 98.94%. Finally, the detection rate for their proposed method was 97.7%, whereas the false positive rate was 4.35% [19].

On the other hand, machine learning algorithms may be vulnerable to adversarial examples [20,21]. In general, adversarial examples can be created by adding subtle perturbations to the training set in order to mislead the machine learning and prevent it from making the right decision. This can also be accomplished through many other techniques, such as but not limited to changing the training set, inserting poisoning attacks into the training set, and creating backdoor attacks during the training phase. Rahim et al. proposed a defense method with two steps in order to identify attacks in regard to adversarial examples in the dataset. They evaluated their method based on three different datasets. They found that the detection rate increased to fifty percent when they used the generative adversarial network (GAN) method [20]. Rahim et al. in [21] also proposed two methods for phone programs in the internet of things (IoT) environment against an adversarial attack to a malware detection system. The first method is a combination of the nearest neighbor (C4C) and ConvNet (CNN) approaches. The second method is Robust-NN. They found that accuracy metrics increased to 94.94% and 96.03% when using their proposal detection method [21].

### 2.4. Deep Learning-Based Detection Techniques

Deep learning is a kind of machine learning. It uses complex techniques that were inspired by the way the human brain works. It can handle a very large number of unstructured data and generate an accurate output without being told which attribute to look for. However, users need to convert unstructured data to structured data, and data take a long time to be processed in machine learning. Waleed et al. in [22] proposed a technique to detect DDoS attacks based on the use of token embedding in order to improve elicited features from session initiation protocol (SIP) texts in VoIP. They also discussed recurrent neural networks (RNNs), which is a deep learning technique that was developed to identify DDoS attacks. Features extracted in their previous stage provided the input for their RNNs. They found that their proposed method could be executed in a short time, and it had high detection accuracy. Wang and Liu proposed a method based on information entropy and deep learning to detect DDoS anomalies in SDNs. This method involves a two-level system of detection. The first is carried out by the controller of SDN to filter suspicious packets in order to increase accuracy by calculating entropy for the important fields of these packets. The second step is to use a deep learning technique—the convolutional neural network (CNN) method—in order to separate benign from malicious traffic. This method can be used in image classification. However, in that study, the authors converted packets to images, then used the CNN to detect DDoS attacks. Finally, they found that the accuracy of their method was 98.98% [23].

### 2.5. Combination-Based Detection Techniques

Recently, researchers used combined techniques to improve the detection of anomalies. For example, they used machine learning techniques and statistical approaches. They found that delays in malicious identification could be appropriately solved. They also found that the identification of low rates could be tackled as well [6]. Researchers can also combine statistical approaches and artificial intelligence to improve the detection results. Daneshgadeh et al. implemented a method that combined two different techniques—statistical and machine learning—in order to detect DDoS attacks. They used a support vector machine (SVM) and two other statistical techniques, Shannon entropy and kernel online anomaly detection (KOAD). They found that Shannon entropy showed improved results when combined with the other two methods in recognizing DDoS attacks and flash events [24]. Ozcelik and Brooks reported a method called CUSUM-Entropy to detect DDoS attacks. They used two statistical methods, consisting of the cumulative algorithm invented by Page and entropy values for traffic features. They calculated entropy features for packets such as the source IP address, the destination IP address, the source port address, and the destination port address. They used both methods in order to improve the detection. Finally, they found that their method had low fall-out and a high true positive rate [25].

## 3. Methodology

Statistical approaches for the detection of anomalies offer a deeper analysis per packet in computer networks. They can observe and analyze information per specific time. Statistical methods for the detection of anomalies are also better than other methods because they are faster than others and work properly in real time [6]. Thus, entropy and SPRT were implemented in this paper to detect DDoS attacks.

### 3.1. Flowchart of ESPRT

Packets have many fields, such as the source IP address, destination IP address, source port address, destination port address, protocol type, flags, header size, connection duration, services, and so on. Flows are a group of packets that have the same specifications such as the source IP address, destination IP address, source port address, destination port address, and protocol type. Incoming packets toward the interfaces of server switches are gathered based on these specifications to form unique flows.

**Figure 2:**
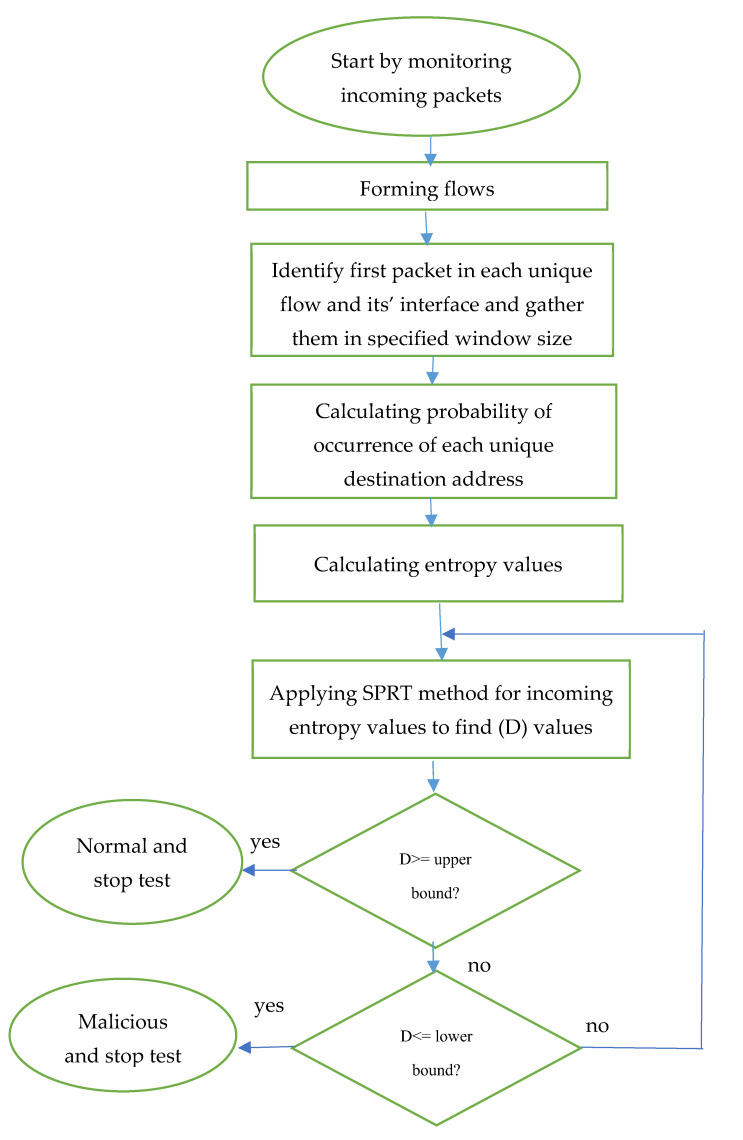
Flowchart for the ESPRT method.

The destination IP addresses of the first packet of each flow are monitored and gathered based on a certain window size. This window size can be determined based on the number of packets or the timeframe. The interface number of the switches (MAC) that allow these flows to pass through will also be recorded in order to locate infected interfaces. These flows will be forwarded towards the entropy stage. The output of the entropy stage is used as the input for the second step, which is SPRT. Finally, a decision can be made for all flows and their interfaces to determine whether they are infected or not at the end of the SPRT detection process. The flow chart for this combination method is shown in Figure 2.

### 3.2. First Phase (Entropy)

The entropy approach was used in the first step in the detection process. Entropy can be calculated to identify the randomness of packets. If entropy values are high, packets are random and, vice versa, when entropy values are low, packets are not random. The randomness of packets is an indicator of benign behavior, as stated in [12].

Destination IP addresses for each flow are grouped in a hash table. These destination IP addresses are inserted into the first column of the table, and the number of their appearances are added to the second column. For example, consider that the first destination IP address packet in each flow is (A), and the number of their appearances is (B). When (A) is new and not available in the hash table, it will be added to the first column, and count one is added to the second column. However, when (A) is available in the table, then counter (B) in the second column of this address is incremented by one, as shown in Equation (1) [12]:

W = {(A_1_, B_1_), (A_2_, B_2_), (A_i_, B_i_), …} (1)

The entropy method depends on the window size, and this window is calculated based on the timeframe or number of packets. Thus, the above equation will be sliding over incoming flows based on window size. Then, the probability (Prob) of an IP destination’s appearance (B) can be calculated for each window as shown in Equation (2) below [12]:

Prob(i) = B(i)/n (2)

where (n) is the total number of packets per window. Finally, entropy values (E) for each window can be calculated based on Equation (3) [12]:

(3) $$E=-\overset{n}{\underset{i=0}{\sum }}\mathrm{prob}\left(i\right)\log_{2}\mathrm{prob}\;\left(i\right)$$

### 3.3. Second Phase (SPRT)

The output for first step (E) is used as an input for the second step in the detection process. The second step is the sequential probability ratio test (SPRT). SPRT was first introduced and developed by Waled in 1947. It is based on mathematical calculation. It has two main components, which are two hypotheses, either the normal hypothesis Y_0_ or the infected hypothesis Y_1_. The switch of the server has many interfaces. The goal is to identify an infected interface, Y_1_, that has been injected with malicious flows and a normal interface, Y_0_, that has normal flows [26].

The SPRT detection method sometimes makes two wrong decisions. This happens when a malicious interface, Y_1_, is falsely identified as a normal interface, Y_0_. This error is called a false negative error. The other mistake happens when a normal interface, Y_0_, is identified as a compromised interface, Y_1_. This error is called a false positive error. Two values were used to tackle these errors. The variable (a) was used to bypass false positive mistakes, and the variable (b) was used to bypass false negative mistakes. The values of these errors should not be greater than these values.

SPRT is designed to monitor a series of entropy observations (E_0_, E_1_, … E_n_) that are coming from the first step. The detection Equation ($D_{n}^{i}$) is a likelihood function of these observations being compromised over these observations being normal, targeting a specific interface (i). Therefore, Equation (4) is expressed as follows [26].

(4) $$D_{n}^{i}=\ln\frac{{\mathrm{prob}\;(E}_{1}^{i},\ldots {,\;E}_{n}^{i}{\;|Y}_{1})}{{\;\mathrm{prob}\;(\;E}_{1}^{i},\;\ldots {,\;E}_{n}^{i}{\;|Y}_{0})}$$

Consider $E_{n}^{i}$ as identically distributed and independent. Then, $D_{n}^{i}$ is expressed as follows [26]:

(5) $$D_{n}^{i}=\overset{m}{\underset{n=1}{\sum }}\ln\frac{{\mathrm{prob}\;(E}_{n}^{i}{|Y}_{1})}{{\;\mathrm{prob}\;(\;E}_{n}^{i}{\;|Y}_{0})}$$

Assuming $E_{n}^{i}$ as Bernoulli distribution values, then [26]:

(6) $$\mathrm{prob}\;(\;0\;\leq {\;E}_{n}^{i}\;\leq \;0.5{|Y}_{0}{)=1-\;\mathrm{prob}\;(\;E}_{n}^{i}\;>\;0.5{|Y}_{0}{)=\mu }_{0}\;$$

(7) $$\;\mathrm{prob}\;(\;0\;\leq {\;E}_{n}^{i}\;\leq \;0.5{|Y}_{1}{)=1-\;\mathrm{prob}\;(\;E}_{n}^{i}\;>\;0.5{|Y}_{1}{)=\mu }_{1}$$

where $\mu_{1}\;$ is larger than $\mu_{0}$ because compromised Ethernet is more likely to be infected with malicious flows. When the values of entropy decreased until they reaches zero, it is more likely that the interface has been injected with malicious flows. When $E_{n}^{i}$ values fall into a range between 0 and 0.5, the probability is that the interface is more likely to be infected with DDoS attacks. However, when $E_{n}^{i}$ values are greater than 0.5, the probability is that the interface is more likely to be normal.

(8) $$D_{n}^{i}=\left\{\begin{array}{c} D_{n-1}^{i}+\ln\frac{{\mathrm{prob}\;(E}_{n}^{i}{|Y}_{1})}{{\mathrm{prob}\;(\;E}_{n}^{i}{\;|Y}_{0})},\;0\;\leq {\;E}_{n}^{i}\;\leq \;0.5\\ D_{n-1}^{i}+\ln\frac{{\mathrm{prob}\;(E}_{n}^{i}\;{|Y}_{1})}{{\mathrm{prob}\;(E}_{n}^{i}{|Y}_{0})}{,\;E}_{n}^{i}\;>\;0.5 \end{array}\right.\;\;$$

By substituting Equations (6) and (7) into Equation (8), the detection function can be expressed as follows (where $D_{0\;}^{i}=0)$:

(9) $$\;\;D_{n}^{i}=\left\{\begin{array}{c} D_{n-1}^{i}+\ln\frac{\mu_{1}}{\mu_{0}},\;0\;\leq {\;E}_{n}^{i}\leq \;0.5\\ D_{n-1}^{i}+\ln\frac{{1-\mu }_{1}}{{1-\mu }_{0}}{,\;E}_{n}^{i}\;>\;0.5 \end{array}\right.$$

(10) $$\left\{\begin{array}{c} A=\ln\frac{b}{\left(1-a\right)}\\ B=\ln\frac{\left(1-b\right)}{a} \end{array}\right.$$

The generated results for $D_{n}^{i}$ are matched with an upper (A) and lower (B) threshold. These two thresholds were computed based on the variables (a) and (b) that were calculated to bypass the false negative rate and false positive rate errors. A and B values were computed as shown in Equation (10). When $D_{n}^{i}$ is larger or equal to the (A) value, Ethernet switch and flow are normal, and testing is stopped. When $D_{n}^{i}$ is smaller or equal to the (B) value, Ethernet switch and flow is considered to be infected and testing is stopped. However, if none of the above two conditions are met, the test continues with another observation.

**Figure 3:**
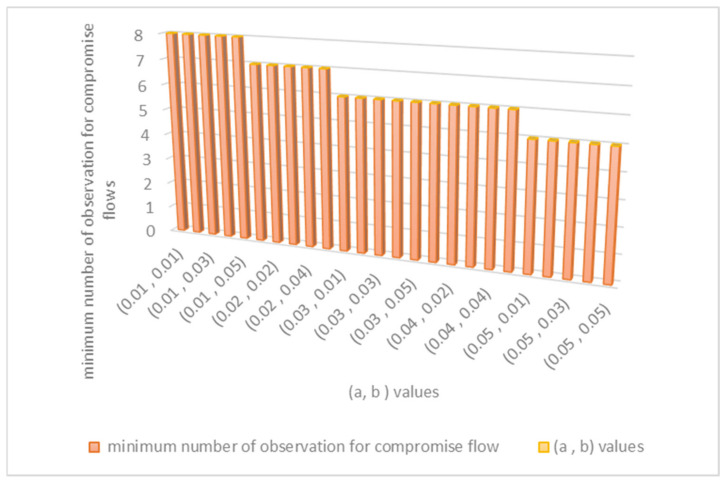
Method of finding the best values for (a) and (b) variables.

The values of variable (a) and variable (b) should be between 0.01 and 0.05 in order to maintain optimum false positive and false negative values for the detection model. Their specific values are chosen based on calculating the minimum number of observations required to identify compromise flows. Figure 3, shown below, explains how to find the best values for (a) and (b). When (a) is 0.05 and (b) is between 0.01 and 0.05, the minimum number of observations of the detection method required to detect malicious flows is only five observations. Therefore, we set (a) to be 0.05 and (b) to be between (0.01 to 0.05) in the evaluation.

## 4. Results

ESPRT was implemented using a Java program and the results are discussed in this section. Other detection methods such as SPRT, Entropy, CUSUM were reimplemented using Java as well. The results were compared with the ESPRT method. The DARPA dataset and confusion matrix were used to evaluate the performance of these detection models [27,28].

### 4.1. Confusion Matrix

The confusion matrix has four main metrics, which are true positive (TP), false positive (FP), true negative (TN), and false negative (FN). From these metrics, other metrics such as accuracy, F1-score, true positive rate (TPR), false positive rate (FPR), true negative rate (TNR), and false negative rate (FNR) can be computed. TPR or sensitivity is the rate of infected flows that were correctly identified as infected flows. TPR can be calculated using the following Equation:

(11) $$\mathrm{TPR}=\frac{\mathrm{TP}}{\mathrm{TP}+\mathrm{FN}}$$

FPR or fall-out is the rate of normal flows that were detected falsely as malicious flows. This metric can be calculated as shown in Equation (12) below:

(12) $$\mathrm{FPR}=\frac{\mathrm{FP}}{\mathrm{FP}+\mathrm{TN}}$$

TNR or specificity is the rate of normal flows that were detected correctly as normal flows. This metric can be calculated as shown in Equation (13).

(13) $$\mathrm{TNR}=\frac{\mathrm{TN}}{\mathrm{TN}+\mathrm{FP}}$$

FNR or miss rate is the rate of infected flows that were falsely recognized as normal flows. It can be calculated as shown below:

(14) $$\mathrm{FNR}=\frac{\mathrm{FN}}{\mathrm{TP}+\mathrm{FN}}$$

Accuracy and F1-scores can be calculated as shown below:

(15) $$\mathrm{Accuracy}=\frac{\mathrm{TP}+\mathrm{TN}}{\mathrm{TP}+\mathrm{TN}+\mathrm{FN}+\mathrm{FP}}$$

(16) $$F1\;\mathrm{score}=2\;\cdot \;\frac{K+\mathrm{TPR}}{K+\mathrm{TPR}}$$

where K is the following equation:

(17) $$K=\frac{\mathrm{TP}}{\mathrm{TP}+\mathrm{FP}}$$

### 4.2. DARPA (Friday_1998 Dataset)

**Figure 4:**
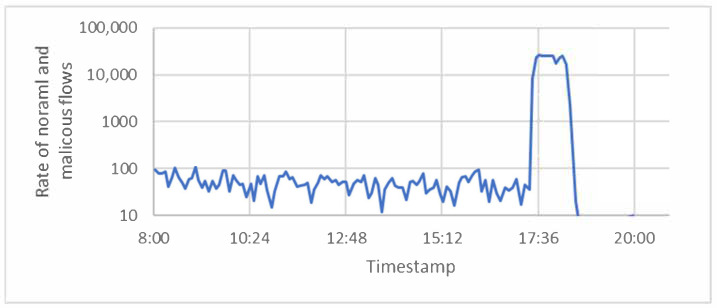
Number of benign and malicious flows per timestamp for the Friday_1998 dataset.

DARPA is based on the Defense Advanced Research Projects Agency. This agency has an impact on different fields of research, such as cybersecurity, communication, and engineering. The MIT Lincoln laboratory, which belongs to this agency, captured datasets that contain DDoS attacks during 1998, 1999, and 2000. The Friday of the 5th week of the DDoS attack in 1998 was one dataset chosen to perform our evaluation. To make it easy, we refer to it as the (Friday_1998) dataset. This dataset has (1,253,312) packets. These packets were gathered into (256,055) flows, as shown in Figure 4. Of these, (1054) were ICMP flows, and (253,397) of these flows were TCP flows. The rest (1596) were UDP flows.

**Figure 5:**
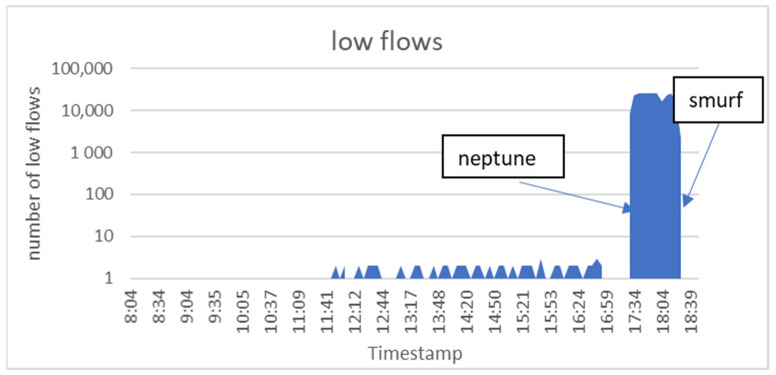
Number of low flows per timestamp for the Friday_1998 dataset.

As mentioned earlier, a flow is a group of packets that have the same specifications. Flows can be classified to low flows and normal flows. The number of packets classified as low flows was smaller than three packets. However, the number of packets classified as normal flows was larger than three packets. This is because attackers were trying to overload servers with a very large number of low flows to consume their resources. Back to the Friday_1998 Dataset, there were a very large number of low flows starting to occur at (17:27:07), and this was due to the ‘neptune’ attack. The ‘smurf’ attack also produced a large number of low flows, starting at (18:00:15), as shown in Figure 5.

### 4.3. Results and Comparison Based on the Friday_1998 Dataset

Both entropy and SPRT methods were used to detect DDoS attacks. However, entropy depends on two elements—threshold and window size. Entropy values have to compare their results with a certain threshold (thr) in order to make decisions about attacks. We chose 0.2 and 1.31 as thresholds because the authors in [12,26] used them to generate their results. Other threshold values were picked randomly to test the efficiency of the detection models. The accuracy and F-scores changed when the threshold values changed, as shown in Table 1 and Table 2. These two tables show values of accuracy and F-scores for different window sizes and threshold values. For example, when the window size is five packets, the accuracy of ESPRT is 0.987. However, the accuracy of entropy with the same window size is 0.986 when the threshold is 0.2, and the accuracy is 0.972 when the threshold is 1.5. The accuracy values of the ESPRT and entropy methods for different window size values are shown in Table 1. Finally, the accuracy and F-scores of entropy improved when entropy was merged with SPRT, as shown in Table 1 and Table 2.

**Figure 6:**
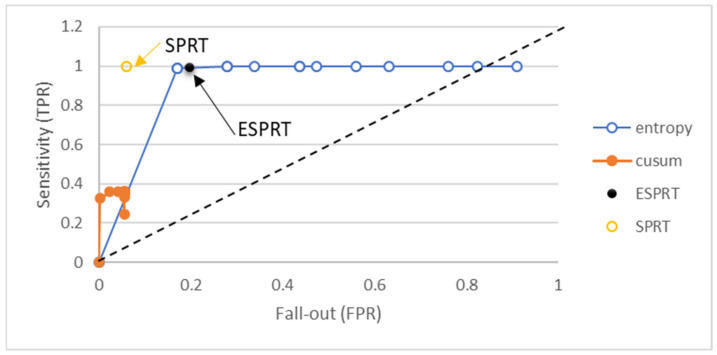
Sensitivity vs. fall-out for different detection methods for the Friday_1998 dataset.

**Figure 7:**
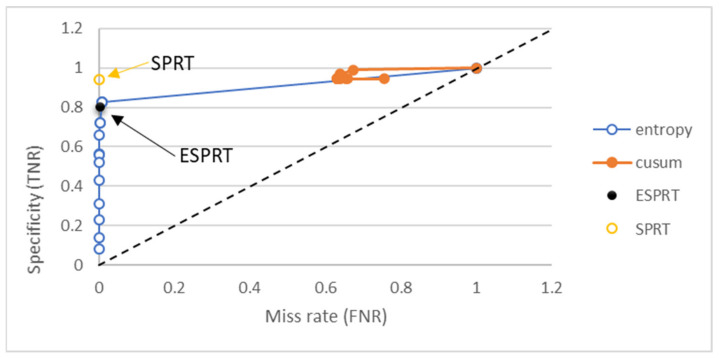
Specificity vs. fall-out for different detection methods for the Friday_1998 dataset.

Confusion metrics were used to explain the differences between ESPRT and other mathematical models. Other methods are entropy, SPRT, and CUSUM. The window size that was used to generate results for the detection methods that require window size was 10 packets. The threshold values for the detection method fell in the range of 0 to 1.9. We used threshold values larger than one because other methods used thresholds greater than one in their detection methods, such as the entropy method. ROC curves, shown in Figure 6 and Figure 7, were drawn to show the differences in terms of TPR, FPR, TNR, and FNR.

When the values of TPR metrics are close to one and the FPR values are close to zero for a certain detection method, this means the detection is good, and vice versa. The value of TPR for ESPRT was 0.995 and for SPRT it was 0.999. However, entropy and CUSUM have different values of TPR and FPR based on changing threshold values. The FPR value for entropy fell in the range of 0.171 and 0.767, based on change in the threshold values. Thus, entropy detection alone generates higher FPRs, and the FPR value of entropy can be reduced when combined with the SPRT method, as shown in Figure 6.

FNR should be closed to zero. FNR was close to zero for ESPRT, entropy, and SPRT. Entropy has different FNR values when the thresholds change, as shown in Figure 7. The TNR value should be close to one for better detection. The TNR for SPRT was 0.940, and ESPRT had a TNR value of 0.802. However, the TNR for entropy fell in the range of 0.08 and 1, based on threshold values. Results for TNR vs. FNR for different methods shown in Figure 7.

### 4.4. Results and Comparison Based on DARPA 2000 Dataset

To further validate the implementation of ESPRT, its results were compared with the results of other researchers’ implemented methods. The DARPA 2000 dataset, used by those researchers, was used to show the differences among these methods in terms of TPR, FPR, FNR, and accuracy. Most methods mentioned in the table have ranges of values, depending on their thresholds or window sizes. We mentioned the minimum and maximum values of the range that were computed by those authors. The average of the range was computed in order to make the comparison easier. For example, the accuracy of ESPRT falls in the range of 89.6% to 93.2% when the window size falls in the range of 5 to 120 packets. The average of the accuracy was 90.5%, which is better than the others, as shown in Table 3.

Finally, the average detection rate for the ESPRT method was 0.929. Cepheli et al. in [32] had a DR average of 0.921. The average detection rate for Chonka et al. in [30] was 0.907, whereas the average DR for Sarmila [31] was 0.166. However, Bista et al. in [17] produced a DR average of 0.591. The DR value for the detection model should reach 1 in order to be perfect. The ESPRT method had a higher value, as mentioned earlier and shown in Table 3. The FPR average for ESPRT was 0.207. This value should be close to 0 for certain detection methods. The FPR values for other researchers can be seen in Table 3 as well. FNR should also be close to zero. The FNR average for ESPRT was 0.069.

### 4.5. Results and Comparison Based on (CIC-DDoS2019) Dataset

In addition, another experiment was carried out using the Canadian Institute for Cybersecurity (CIC-DDoS2019) dataset [33] in order to reflect the security assurance of entropy-based methods. The system topology, kinds of DDoS attacks, traffic volume, and other important information are all available in [33]. The datasets were captured for two days. These datasets contain many files per day. Random samples were picked, and an experiment was run on ESPRT. Table 4, presented below, shows the confusion matrix results for ESPRT based on the CIC-DDoS2019 dataset.

### 4.6. Scalability of ESPRT

**Figure 8:**
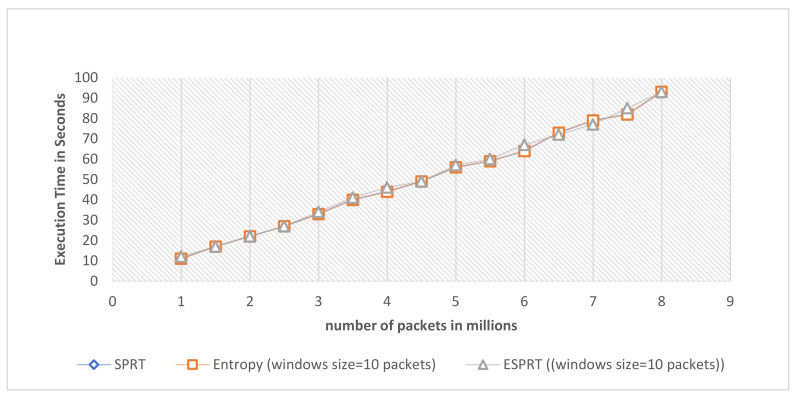
Execution time in seconds per specified number of packets for ESPRT, SPRT, and entropy methods.

To measure the scalability of the ESPRT method, the DARPA 1999 dataset was used. DARPA 1999 has multiple domains that can communicate with each other. The ESPRT, SPRT, and entropy detection models can monitor and detect DDoS attacks on all available domains in the network. This helps to measure scalability on multiple domains. The execution time in seconds for ESPRT, SPRT, and entropy methods was calculated per specified packet numbers, as shown in Figure 8. The memory RAM size of the device that was used to conduct the experiment is 16 GB. The CPU was 2.70 GHZ. The values of execution time for these three models were close to each other. In other words, ESPRT shows scalability, as shown in Figure 8.

## 5. Conclusions and Future Works

DDoS attacks are very dangerous. They can bring down targeted servers and prevent users from accessing their services. Statistical approaches for the detection of anomalies provide perfect tools to analyze and observe flows. They are faster than other techniques and can be performed in real time. Therefore, the ESPRT method was implemented in order to identify DDoS attacks. ESPRT is a combination of two detection methods—the entropy and SPRT methods. DARPA databases were used to evaluate their implementation, and confusion metrics were used to compare ESPRT results with those of other methods.

In addition, using both entropy and SPRT removed the uncertainty involved in the entropy threshold, and improved the entropy results. The accuracy and F-score values for entropy changed when the threshold values changed. The results of other metrics also changed when the threshold values changed, such as the true positive rate (TPR), false positive rate (FPR), true negative rate (TNR), and false negative rate (FNR).

Finally, ESPRT showed an accuracy value of 0.995 when the value of the window size was 50 and 75 packets. It had an F-score of 0.997 when the value of the windows size was 50 and 75. It had a true positive rate of 0.995, and a true negative rate of 0.802. However, it had a false positive rate of 0.197, and a false negative rate of 0.004. ESPRT also showed an average accuracy of 90.5% and a detection rate of 0.929 when applied on the DARPA 2000 dataset. Finally, ESPRT was scalable onto multiple-domain topology.

For the sake developing of this study in the future, we are going to dig deeper into the adversarial example field. Adversarial examples cause machine learning techniques to make wrong decisions. We will evaluate the detection rate of ESPRT method when encountering adversarial examples. Finally, the development of methods for the extraction of other features from packets and their effects on the ESPRT method will be also explored.

## Figures and Tables

**Table 1:** Accuracy of ESPRT and entropy for different window sizes and thresholds.

Window Size	Accuracy of ESPRT Detection	Accuracy of Entropy Detection				
		(Thr = 0.2)	(Thr = 0.5)	(Thr = 1)	(Thr = 1.31)	(Thr = 1.5)
5	0.987	0.986	0.986	0.980	0.979	0.972
10	0.990	0.986	0.989	0.984	0.980	0.978
25	0.988	0.991	0.992	0.989	0.986	0.984
50	0.995	0.987	0.994	0.992	0.990	0.989
75	0.995	0.986	0.994	0.993	0.991	0.989
100	0.993	0.984	0.994	0.993	0.992	0.990

**Table 2:** F-score of ESPRT and entropy for different window sizes and thresholds.

Window Size	F-Score of ESPRT Detection	F-Score of Entropy Detection				
		(Thr = 0.2)	(Thr = 0.5)	(Thr = 1)	(Thr = 1.31)	(Thr = 1.5)
5	0.993	0.993	0.993	0.990	0.989	0.986
10	0.994	0.993	0.994	0.991	0.990	0.989
25	0.994	0.995	0.996	0.994	0.993	0.991
50	0.997	0.993	0.997	0.996	0.994	0.994
75	0.997	0.993	0.997	0.996	0.995	0.994
100	0.996	0.992	0.996	0.996	0.995	0.994

**Table 3:** Comparison with other approaches based on the DARPA 2000 dataset.

Method	Accuracy	DR or TPR	FPR	FNR	Window Size	Thr
ESPRT	89.6% to 93.2% (average = 90.5%)	0.914 to 0.957 (average = 0.929)	0.154 to 0.270 (average = 0.207)	0.042 to 0.078 (average = 0.069)	Window size range between 5 and 120 packets	No need for Thr in this method.
Real-time DDoS attack [29]	22% to 100% (average = 90.3%)	NA to 1 (They mentioned the maximum value only)	0 to NA	0.18 to NA	No need for window size in this method.	Threshold values range between 0.1 and 0.9 (22% when thr = 0.3, 70% when thr = 0.4, 100% when thr = 0.5 or higher).
Chaos theory [30]	NA	0.88 to 0.94 (average = 0.907)	0.05 to 0.45 (average = 0.233)	NA	No need for window size in this method.	Threshold values range between 0.1 to 0.9
HCA with Labelling [17,31]	41% to 95% (average = 72.42%)	0.048 to 0.383 (average = 0.166)	0.167 to 0.523 (average = 0.237)	NA	Window size range between 10 and 120 packets	No need for Thr in this method.
HCA with Naïve Base Classification [17]	58% to 100% (average = 86.73)	0.560 to 1 (average = 0.591)	0 to 0.352 (average = 0.119)	NA	Window size range between 10 and 120 packets	No need for Thr in this method.
H-IDS [32]	NA	0.921	0.18	NA	No need for window size in this method.	No need for Thr in this method.

NA: not available, DR: detection rate, TPR: true positive rate, FPR: false positive rate, FNR: false negative rate, Thr: threshold.

**Table 4:** The confusion matrix results for ESPRT, based on the CIC-DDoS2019 dataset.

Dataset	Accuracy	F1-Score	DR or TPR	FPR	FNR
**Sample#1 (SAT-03-11-2018_0137)**	0.997	0.998	0.997	0	0.002
**Sample#2 (SAT-03-11-2018_010)**	0.997	0.998	0.997	0	0
**Sample#3 (SAT-03-11-2018_030)**	0.975	0.987	0.975	0.287	0.024
**Sample#4 (SAT-03-11-2018_070)**	0.974	0.997	0.994	0.320	0.005
**Sample#5 (SAT-03-11-2018_0110)**	0.993	0.996	0.993	0.1	0.006

## Data Availability

The data obtained during the study are publicly available online. These datasets belong to the MIT Lincoln Laboratory. The 5th week of the DDoS attack in the 1998 dataset was one dataset chosen for evaluation, and it is available online at https://archive.ll.mit.edu/ideval/data/1998/training/week5/index.html (accessed on 2 July 2021). Another dataset is DARPA 2000, and it is available online at https://archive.ll.mit.edu/ideval/data/2000/LLS_DDOS_2.0.2.html (accessed on 2 July 2021). Another dataset is (CIC-DDoS2019) Dataset, and it is available online at https://www.unb.ca/cic/datasets/ddos-2019.html (accessed on 29 August 2021).
